# Secondary-Heteroatom-Doping-Derived Synthesis of N, S Co-Doped Graphene Nanoribbons for Enhanced Oxygen Reduction Activity

**DOI:** 10.3390/nano12193306

**Published:** 2022-09-23

**Authors:** Bing Li, Tingting Xiang, Yuqi Shao, Fei Lv, Chao Cheng, Jiali Zhang, Qingchao Zhu, Yifan Zhang, Juan Yang

**Affiliations:** School of Materials Science and Engineering, Jiangsu University, Zhenjiang 212013, China

**Keywords:** secondary-heteroatom-doping, graphene nanoribbons, unzipping, oxygen reduction reaction

## Abstract

The rareness and weak durability of Pt-based electrocatalysts for oxygen reduction reactions (ORRs) have hindered the large-scale application of fuel cells. Here, we developed an efficient metal-free catalyst consisting of N, S co-doped graphene nanoribbons (N, S-GNR-2s) for ORRs. GNRs were firstly synthesized via the chemical unzipping of carbon nanotubes, and then N, S co-doping was conducted using urea as the primary and sulfourea as the secondary heteroatom sources. The successful incorporation of nitrogen and sulfur was confirmed by elemental mapping analysis as well as X-ray photoelectron spectroscopy. Electrochemical testing revealed that N, S-GNR-2s exhibited an E_onset_ of 0.89 V, E_1/2_ of 0.79 V and an average electron transfer number of 3.72, as well as good stability and methanol tolerance. As a result, N, S-GNR-2s displayed better ORR property than either N-GNRs or N, S-GNRs, the control samples prepared with only a primary heteroatom source, strongly clarifying the significance of secondary-heteroatom-doping on enhancing the catalytic activity of carbon-based nanomaterials.

## 1. Introduction

The World Energy Outlook 2021 report, released by the International Energy Agency (IEA), pointed out that the growth speed of new energy power is not fast enough to support the goal of net zero emissions by 2050 [1]. Therefore, new energy conversion and storage devices (such as fuel cells [2,3,4], metal air batteries [5,6,7], lithium-ion batteries [8,9,10], supercapacitors [11,12,13], etc.) continue to draw researchers’ attention. A fuel cell is a device that can convert the chemical energy of a fuel and an oxidizer directly into electric energy with the help of catalysts. It is an environmentally friendly technology with a high energy density and conversion rate [14].

The oxygen reduction reaction (ORR) is a vital electrochemical process which occurs at the cathode side of fuel cells. The sluggish kinetics of ORRs result in serious cathode polarization and energy loss, making them one of the major challenges of the large-scale implementation and commercialization of fuel cells [15,16]. To date, Pt-based nanomaterials still play an important role in catalyzing ORRs as they are regarded as the most efficient catalysts [17]. However, numbers of research works have been conducted on exploring alternative ORR electrocatalysts with a low price and outstanding catalytic performance due to the prohibitive cost, poor stability and methanol tolerance of commercial Pt-based catalysts [18].

Carbon nanomaterials are a hot topic in recent years because of their special structure, physical and chemical properties, and thus their wide range of applications [19,20]. Heteroatoms (e.g., nitrogen, sulfur, boron, phosphorus) doped into carbon nanomaterials could induce electron modulation, rendering the charge distribution and facilitating the adsorption of O_2_ to enhance the ORR activity [21]. Nitrogen has an atomic size similar to that of carbon but a different electron configuration, such that nitrogen atoms can change carbon nanomaterials’ electronic structures while minimizing the lattice mismatch [22]. N-doped materials, including carbon nanotubes (CNTs) [23,24,25], graphene [26,27,28] as well as porous carbons [29,30,31], have been studied for years in order to regulate nanomaterials’ electronic structures and some other properties. In addition, sulfur has also been confirmed to have a beneficial effect on the ORR activity of carbon nanomaterials. The electronegativity of sulfur atoms is similar to that of carbon atoms (C = 2.55, S = 2.58), leading to the change in the spin density of carbon atoms as well as more defect sites [32]. These characteristics make sulfur the second-most efficiently doped element after nitrogen. Given the facts above, the co-doping of nitrogen and sulfur atoms has the potential to further regulate the electronic structure of the adjacent carbon atoms, resulting in a synergistic effect on enhancing ORR properties [33].

Graphene nanoribbons (GNRs) have drawn growing research attention for their unique structure with a large length–width ratio and abundant edge content [34]. They can be regarded as the products of the longitudinal unzipping of CNTs. In addition, GNRs show characteristics such as high chemical stability, low weight, decent specific surface area and low price, making themselves possible to be applied in the field of electrocatalysis [35]. In our previous work, N-doped GNRs (N-GNRs) were successfully prepared and electrochemical tests were conducted [36]. The synergistic effect between N-doping and carbon edges on catalyzing ORRs has been demonstrated, and as a result, as-obtained catalysts exhibited superior ORR property. On the basis of the discussion above, if an additional heteroatom of sulfur is introduced into N-GNRs, the obtained new catalyst can take full advantage of the synergy among the edge structure, nitrogen doping and sulfur doping, and thus the ORR catalytic activity is expected to be further improved [37,38,39].

Herein, GNRs were firstly prepared via the chemical unzipping of multi-walled CNTs, and N, S co-doped GNRs (N, S-GNR-2s) were obtained by high-temperature annealing with urea as the primary and sulfourea as the secondary heteroatom precursors. Structural characterizations of the sample have verified the successful incorporation of nitrogen and sulfur. N, S-GNR-2s showed better ORR activity than other GNR-based samples doped only with a primary heteroatom source, demonstrating the importance of secondary-heteroatom-doping. As a result, N, S-GNR-2s mainly displayed a 4-electron catalytic pathway towards an ORR, rendering themselves potential ORR electrocatalyst alternatives for the application on fuel cells.

## 2. Materials and Methods

### 2.1. Synthesis of N-GNRs, N, S-GNRs and N, S-GNR-2s

GNRs were obtained by the unzipping of multi-walled CNTs (full details are given in the Supplementary Materials) [40]. GNRs and urea (mass ratio of 1:20) were mixed and grinded in an agate grinding bowl [41,42]. The mixture was slowly transferred into a quartz boat and put in a tube furnace, and then underwent thermal annealing in an Ar atmosphere at 900 °C for 2 h to obtain N-GNRs. N-GNRs and sulfourea (mass ratio of 1:20) followed the same steps above but pyrolyzed at different temperatures (800 °C, 900 °C and 1000 °C) for 2 h to obtain N, S-GNR-2s.

To synthesize the control sample of N, S-GNRs, GNRs and sulfourea (1:20) were also mixed and grinded in a crucible and then annealed in Ar at 900 °C for 2 h.

### 2.2. Structural Characterization

Scanning electron microscopy (SEM, JEOL JSM-7800F; Tokyo, Japan) and transmission electron microscopy (TEM, JEOL JEM-2100; Tokyo, Japan) were applied to observe the morphology of the catalysts. Element mapping analysis was detected to know the distribution of the elements. X-ray photoelectron spectroscopy (XPS, ESCALAB 250Xi; Thermo Fisher, Waltham, MA, USA) was conducted to analyze the bond configuration of the elements. Raman spectra were collected to monitor the defect level of the samples. All instrument parameters are shown in the Supplementary Materials.

### 2.3. Electrochemical Characterization

A 2 mg catalyst was uniformly dispersed in a 1 mL mixed solvent of isopropanol and nafion (19.88:0.12) to form a homogeneous ink. Then, a 20 μL catalyst ink was loaded onto a rotating disk electrode (RDE) or rotating ring-disk electrode (RRDE) and dried. A concentration of 0.1 M of KOH was used as the electrolyte with O_2_ or N_2_ saturation.

All of the electrochemical characterization was performed in a standard three-electrode cell (a Pt wire as the counter electrode, an Ag/AgCl as the reference electrode and an RDE or RRDE as the working electrode). The potentials in this study were converted according to the equation of E_vs. RHE_ = E_vs. Ag/AgCl_ + (0.059 pH + 0.197) V [43]. Full details of the electrochemical characterization including cyclic voltammetry (CV), RDE and RRDE measurements are given in the Supplementary Materials.

## 3. Results and Discussion

**Figure 1:**
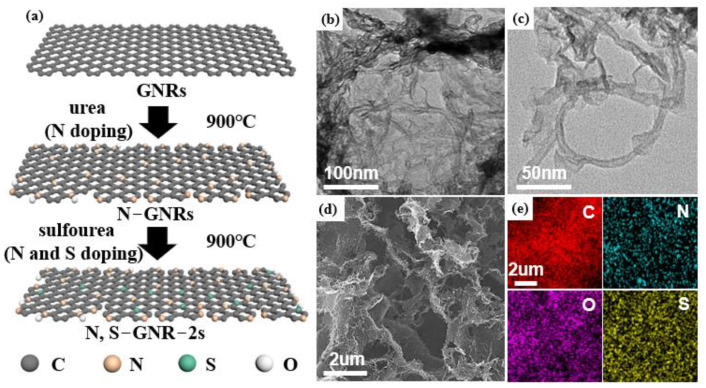
(**a**) The synthesis process of N, S-GNR-2s. (**b**,**c**) TEM images of N, S-GNR-2s. (**d**) The SEM image and (**e**) the corresponding EDS element mappings of N, S-GNR-2s showing the distribution of C, N, O and S.

Figure 1a illustrates the fabrication process of N, S-GNR-2s. Urea was utilized as the primary heteroatom source to form the N-GNRs via thermal annealing. Then, sulfourea was used as the secondary heteroatom source to incorporate sulfur as well as additional nitrogen to form N, S-GNR-2s. TEM and SEM were applied to observe the morphology of our sample. After chemical oxidation and N, S co-doping, the ribbon structure can be clearly seen (Figure 1b,c), indicating that the CNTs were successfully unzipped and GNRs were formed. However, some side walls of CNTs still existed, implying that the oxidative unzipping was not 100% complete. An SEM image of N, S-GNR-2s is shown in Figure 1d, and the corresponding energy dispersive spectrometer (EDS) elemental mapping proved that nitrogen and sulfur were successfully doped and uniformly distributed on N, S-GNR-2s (Figure 1e).

**Figure 2:**
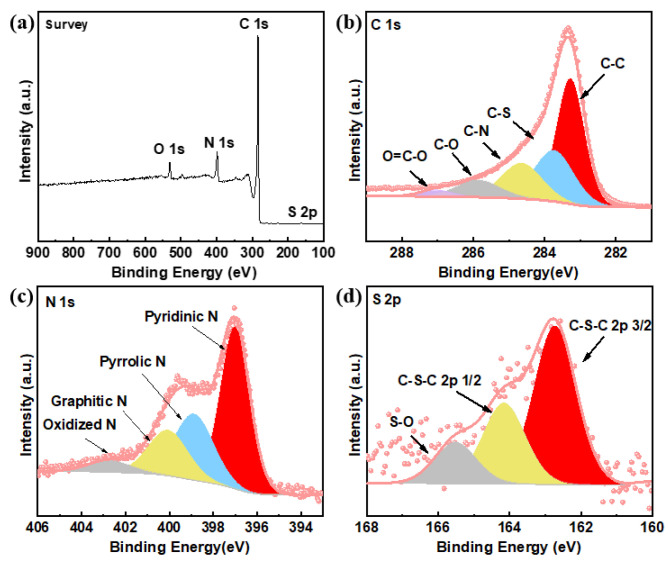
(**a**) The XPS survey, (**b**) C 1s, (**c**) N 1s and (**d**) S 2p spectra of N, S-GNR-2s.

A variety of structural characterizations were used to examine the physical properties of our samples. The existence of C, N, O and S was detected by the XPS analysis of N, S-GNR-2s (Figure 2a), further proving the successful incorporation of nitrogen and sulfur. In the C 1s spectrum, C–C, C–S, C–N, C–O and O = C–O bonds could be directly observed (Figure 2b), demonstrating that parts of N, S-GNR-2s were oxidized with N and S co-doping. The N 1s spectrum can be divided into four peaks: pyridinic N, pyrrolic N, graphitic N and oxidized N (Figure 2c). Since the GNRs possessed abundant edge structures and pyridine N was located at the edges of the carbon materials, the content of pyridine N was high (48.78%) as expected. Figure 2d is the S 2p spectrum of N, S-GNR-2s, in which the fitting peaks of 162.8 eV (S 2p_3/2_) and 163.9 eV (S 2p_1/2_) are characteristic peaks of thiophene S with different spin orbital coupling. There is also a small peak of S–O at 167.1 eV. Generally, thiophene S is considered as the key coordination type to enhance ORR performance [44,45]. The results confirmed that N and S replaced a small amount of C in the material and acted as dopants, which was beneficial to improving the ORR performance of the catalysts.

**Figure 3:**
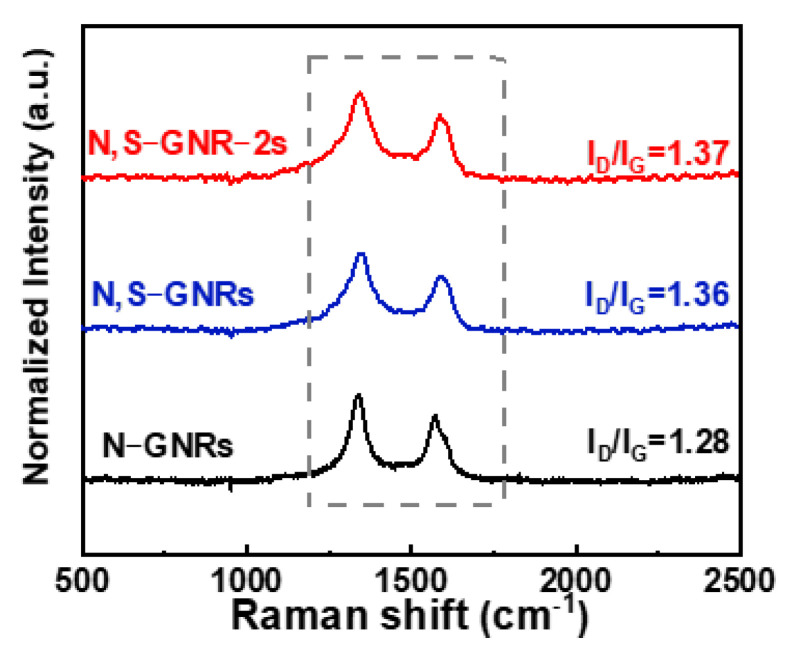
The Raman spectra of N-GNRs, N, S-GNRs and N, S-GNR-2s.

The D band (located at ~1360 cm^−1^) and G band (located at ~1580 cm^−1^) are shown in the Raman spectra of N-GNRs, N, S-GNRs and N, S-GNR-2s (Figure 3). The I_D_/I_G_ ratio of N-GNRs was calculated to be 1.28, owing to N-doping and the formation of the porous structure caused by the release of gas generated during the thermal decomposition of urea. Compared with the N-GNRs, both the N, S-GNRs and N, S-GNR-2s had more defects caused by additional S-doping. As a result, the I_D_/I_G_ ratios of the N, S-GNRs and N, S-GNR-2s were increased to 1.36 and 1.37, respectively [46].

**Figure 4:**
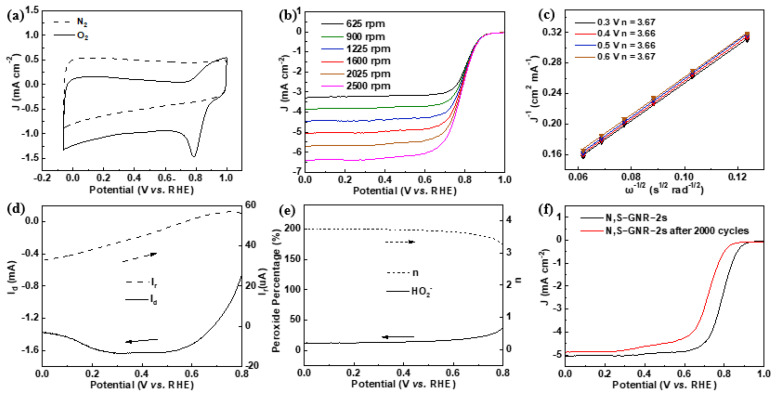
Electrocatalytic property tests of N, S-GNR-2s. (**a**) The CV test in 0.1 M KOH solution. (**b**) The RDE test at rotating speeds from 625 to 2500 rpm. (**c**) K–L plots based on the RDE test. (**d**) RRDE curves at a rotating speed of 1600 rpm. (**e**) The calculated HO_2_^−^ yield and electron transfer number. (**f**) The stability test before and after 2000 cycles.

Electrochemical tests of N, S-GNR-2s were carried out in an alkaline medium of 0.1 M KOH. Three samples of N, S-GNR-2s with different thermal annealing temperatures (800 °C, 900 °C and 1000 °C) were examined, among which, N, S-GNR-2s at 900 °C showed the best ORR activity (Figure S1). For this reason, this sample was selected in the following experiments for the performance analysis. Figure 4a displays CV curves of N, S-GNR-2s in O_2_ and N_2_-saturated solution. No evident peaks appeared when tested in N_2_-saturated solution, whereas a distinct ORR peak in O_2_-saturated solution was clearly observed at 0.80 V, suggesting good ORR catalytic activity for N, S-GNR-2s. RDE measurement was carried out at different rotating speeds from 625 to 2500 rpm (Figure 4b), and Koutecky–Levich (K–L) plots are correspondingly displayed in Figure 4c. The electron transfer number was extracted to be 3.66~3.67, ranging from 0.3~0.6 V. In addition, RRDE testing was further performed in order to accurately detect the generation of peroxide (HO_2_^−^) during the reaction (Figure 4d). The average HO_2_^−^ yield was measured and calculated to be about 13.69%, and the average electron transfer number was calculated to be about 3.72 from 0 to 0.8 V (Figure 4e), in correspondence with the result in Figure 4c. The electrocatalytic properties tested by RDE and RRDE distinctly verified that N, S-GNR-2s mainly displayed a 4-electron catalytic pathway towards an ORR. RDE curves at 1600 rpm before and after 2000 CV cycles with a scan rate of 100 mV s^−1^ and potential range of 0.56~0.96 V are shown to evaluate the stability of N, S-GNR-2s (Figure 4f). The diffusion-limited current density after cycling stayed almost unchanged, and the half-wave potential (E_1/2_) slightly shifted from 0.79 V to 0.71 V, demonstrating the decent ORR stability of our catalysts.

**Figure 5:**
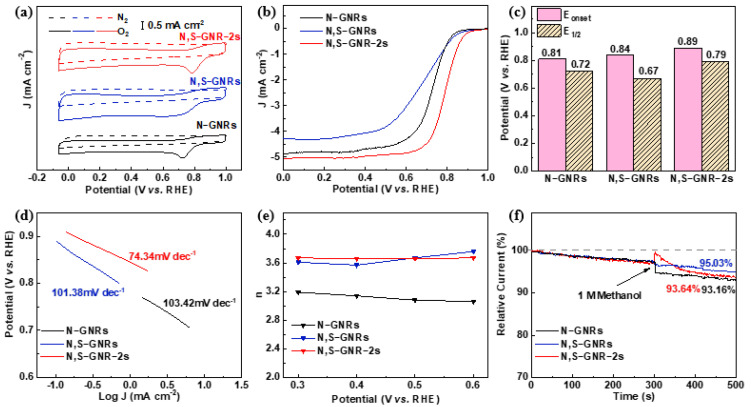
Electrocatalytic property tests of N-GNRs, N, S-GNRs and N, S-GNR-2s. (**a**) The CV test in 0.1 M KOH. (**b**) The RDE test at a rotating speed of 1600 rpm. (**c**) The corresponding values of E_onset_ and E_1/2_. (**d**) The corresponding Tafel plots based on the RDE tests. (**e**) The values of electron transfer number (n) at certain potentials. (**f**) The methanol tolerance test.

In order to compare the catalytic performance of different samples (N-GNRs, N, S-GNRs and N, S-GNR-2s) and verify the significance of secondary-heteroatom-doping, various electrochemical analyses were carried out. A CV test was performed, and the curves were recorded and plotted together (Figure 5a). The CV curve of N, S-GNR-2s in O_2_ exhibited a more positive oxygen reduction peak than those of the other two samples, revealing the superior catalytic activity of N, S-GNR-2s. Figure 5b shows the RDE curves at 1600 rpm of the three samples, and the corresponding values of onset potential (E_onset_) and E_1/2_ were plotted in Figure 5c. Among all the samples, N, S-GNR-2s showed the highest E_onset_ of 0.89 V and E_1/2_ of 0.79 V along with a diffusion-limited current density of up to 5.06 mA cm^−2^, while N-GNRs displayed an E_onset_ of 0.81 V and E_1/2_ of 0.72 V, and N, S-GNRs displayed an E_onset_ of 0.84 V and E_1/2_ of 0.67 V. Tafel plots of the catalysts are displayed in Figure 5d on the basis of the RDE test. N, S-GNR-2s presented a much lower Tafel slope of 74.34 mV dec^−1^ than other catalysts, suggesting the most desirable ORR activity of N, S-GNR-2s. The values of electron transfer number are shown in Figure 5e on the basis of the RDE tests (Figure 4, Figures S2 and S3). N, S-GNR-2s exhibited similar but more steady values than N, S-GNRs, and both of their performances surpassed that of N-GNRs. Chronoamperometric measurement was conducted to test the methanol tolerance of these samples at 1600 rpm at 0.5 V (Figure 5f). When 1 M methanol was added to the test system, they exhibited different levels of performance decay. Interestingly, N, S-GNRs were found to display a high current retention of 95.03% after 500 s, slightly higher than 93.64% for N, S-GNR-2s and 93.16% for N-GNRs, certifying a good methanol tolerance property for all the catalysts. We compared the electrocatalytic property of N, S-GNR-2s with some relevant studies (Table S1), and the results revealed that N, S-GNR-2s showed competitive or even better performance compared to the reported works.

To sum up the electrochemical performance above, interesting conclusions can be drawn. Firstly, comparing the performance of N, S-GNR-2s with N-GNRs and N, S-GNRs, the significance of secondary-heteroatom-doping was remarkably certified. Both N-GNRs and N, S-GNRs were synthesized using only a primary heteroatom source, and thus they presented inferior activity to N, S-GNR-2s with secondary-heteroatom-doping. In order to understand the effect of secondary-heteroatom-doping, we further conducted the XPS analysis of N-GNRs and N, S-GNRs. As shown in Figure S4 and Table S2, N, S-GNR-2s displayed a N content of 10.93%, much higher than that of either N-GNRs (5.43%) or N, S-GNRs (5.67%). Thus, it can be concluded that the heteroatom content was greatly increased by secondary-heteroatom-doping, leading to more reaction sites and thus improved ORR property. Secondly, the performance difference between N, S-GNR-2s and N-GNRs also proved that the synergistic effect between N and S induced by N, S co-doping contributed to the enhancement of catalytic activity. Consequently, the highest electrocatalytic performance was obtained for our well-designed catalyst of N, S-GNR-2s.

## 4. Conclusions

In summary, N, S-GNR-2s were prepared via the N, S co-doping of GNRs using urea and sulfourea as the primary and secondary heteroatom precursors, respectively. The ribbon structure of N, S-GNR-2s was confirmed by TEM observation, suggesting the successful unzipping of CNTs. The introduction of both nitrogen and sulfur was verified by various structural characterizations. Electrochemical testing showed that N, S-GNR-2s exhibited an E_onset_ of 0.89 V, E_1/2_ of 0.79 V and an average electron transfer number of 3.72, as well as good stability and methanol tolerance. As a result, N, S-GNR-2s revealed better ORR property than N-GNRs and N, S-GNRs, demonstrating that secondary-heteroatom-doping contributes greatly to the improvement of electrocatalytic performance for carbon nanomaterials.

## Data Availability

The data presented in this study are available upon request from the corresponding author.

## Supplementary Materials

The following supporting information can be downloaded at: https://www.mdpi.com/article/10.3390/nano12193306/s1, Figure S1. Electrochemical characterizations of N, S-GNR-2s synthesized at different temperatures in 0.1 M KOH. Figure S2. Electrochemical characterizations of N-GNRs. Figure S3. Electrochemical characterizations of N, S-GNRs. Figure S4. An XPS survey of N-GNRs, N, S-GNRs and N, S-GNR-2s. Table S1. An electrocatalytic property comparison between our work and some relevant studies. Table S2. The elemental contents of N-GNRs, N, S-GNRs and N, S-GNR-2s based on XPS analysis in Figure S4. References [43,47,48,49,50,51,52,53,54,55] are cited in the supplementary materials.
