# Supplementary material for: Secondary-Heteroatom-Doping-Derived Synthesis of N, S Co-Doped Graphene Nanoribbons for Enhanced Oxygen Reduction Activity
Source: Nanomaterials (Basel). 2022 Sep 23;12(19):3306. doi: 10.3390/nano12193306 (PMC9565512; doi:10.3390/nano12193306)
Supplement: Supplementary file 1 [file nanomaterials-12-03306-s001.zip › nanomaterials-1906131-supplementary.pdf]

Supplementary Materials

Secondary−Heteroatom−Doping−Derived Synthesis of N, S Co−Doped Graphene Nanoribbons for Enhanced Oxygen Reduction Activity

Bing Li[†], *Tingting Xiang*[†], *Yuqi Shao, Fei Lv, Chao Cheng, Jiali Zhang, Qingchao Zhu, Yifan Zhang and Juan Yang**

School of Materials Science and Engineering, Jiangsu University, Zhenjiang 212013, China

*Correspondence: yangjuan6347@ujs.edu.cn

[†] These authors contributed equally to this work.

Materials and reagents

Multi−walled CNTs were purchased from Suzhou Tanfeng Graphene Technology Co., Ltd. H_2SO_4 (AR, 98%), HCl (AR, 38%), H_2O_2 (AR, 30%), KOH (AR, 99%), $KMnO_4$ (AR, 99.5%) , urea (AR, 98.5%) and sulfourea (AR, 99%) were obtained from Sinopharm Chemical Reagent (SCR, Shanghai, China). All reagents were used as received without further purification.

Synthesis of GNRs

300 mg of commercial multi−walled CNTs were the raw materials and mixed with 72 mL of 98% H_2SO_4 in a small beaker. The mixture was stirred for 30 min and dispersed in the ultrasonic machine for 10 min. 1.5 g of $KMnO_4$ was slowly added into the mixture, and the beaker was placed in a larger beaker with ice water. The mixture was put in an oil−bath pan and the temperature was kept at 65 °C for 1 h. The solution was then poured into 500 mL of ice, followed by the addition of 18 mL of H_2O_2. The reaction lasted for 10 min, and GNRs were obtained after filtration, rinsing and freeze drying.

Structural characterization

The microstructure of samples was observed by scanning electron microscopy (SEM, JEOL JSM−7800F; Tokyo, Japan), with an acceleration voltage of 5 kV, and by high−resolution transmission electron microscopy (TEM, JEOL JEM−2100; Tokyo, Japan) working at 120 kV. Element mapping analyses were detected using a JEOL JSM−2100F microscope at an accelerating voltage of 15 kV. X−ray photoelectron spectroscopy (XPS, ESCALAB 250Xi; Thermo Fisher, Waltham, MA, USA), with the

excitation source of Mg Kα, was used to analyze the chemical composition and bond configuration, and the detection sensitivity of XPS was at 0.1%. Raman spectra were collected using a Raman spectrometer (Thermo Fisher, Waltham, MA, USA) with a 532 nm wavelength of laser light at 5 mW).

Electrochemical characterization

Electrochemical characterization of the catalysts was performed in a standard three−electrode cell using an electrochemical workstation (CHI760E; Shanghai Chen Hua Medical Instruments Co., Ltd., Shanghai, China). A Pt wire counter electrode and an Ag/AgCl reference electrode in saturated KCl solution were used. A rotating disk electrode (RDE; PIN USA, Glendale, CA) with a glassy carbon disk (5 mm in diameter) was used as the working electrode for cyclic voltammetry (CV) and RDE measurement. A rotating ring−disk electrode (RRDE; PIN USA, Glendale, CA) with a glassy carbon disk (area of 0.2475 cm^2) and a Pt ring (area of 0.1866cm^2) was used as the working electrode for RRDE measurement. All potentials measured against an Ag/AgCl electrode in this study were converted to potentials vs. reversible hydrogen electrode (RHE) according to $E_{vs. RHE} = E_{vs. Ag/AgCl} + (0.059\ pH + 0.197)$ V.

Catalysts (2 mg) were dispersed in a 1 mL mixed solvent of isopropanol and nafion (19.88:0.12) for at least 2 h of sonication to form a homogeneous ink. Then, the catalyst ink (20 μL) was loaded onto an RDE or RRDE and dried overnight at room temperature. An electrolyte of 0.1 M KOH was saturated with oxygen or nitrogen by bubbling O_2 or N_2 for 30 min before each experiment. Prior to all the measurements, the deposited

catalyst was electrochemically cleaned in N_2–saturated 0.1 M KOH for 20 CV cycles at a scan rate of 50 mV s^{-1}.

For CV measurement, the working electrode was cycled between 1.17 ~ −0.06 V in O_2– or N_2–saturated KOH solution at 50 mV s^{-1}. RDE measurement was conducted from 0.96 ~ −0.06 V at rotation speeds from 625 to 2500 rpm in O_2–saturated 0.1 M KOH at 10 mV s^{-1}. A flow of O_2 was maintained over the electrolyte during the recording of the RDE in order to ensure its continuous gas saturation. Koutecky–Levich (K–L) plots (J^{-1} vs. $\omega^{-1/2}$) were analyzed at various electrode potentials. The slopes of their linear fit lines were used to calculate the number of electrons transferred (n) on the basis of the K–L equation [43]:

$$\frac{1}{J} = \frac{1}{J_K} + \frac{1}{J_L} = \frac{1}{J_K} + \frac{1}{0.62nFC_0D_0^{2/3}v^{-1/6}\omega^{1/2}}$$

where J is the measured current density, J_K and J_L are the kinetic and diffusion–limiting current densities, ω is the angular velocity, n is the electron transfer number, F is the Faraday constant (96485 C mol^{-1}), C_0 is the concentration of dissolved O_2 (1.2×10^{-6} mol cm^{-3}), D_0 is the diffusion coefficient of O_2 (1.9×10^{-5} cm^2 s^{-1}) and v is the kinematic viscosity of the electrolyte (0.01 cm^2 s^{-1}). For the Tafel plots, the kinetic current was calculated from the mass–transport correction of the RDE by:

$$J_K = \frac{J_L \times J}{J_L - J}$$

RRDE measurement was conducted at a rotation speed of 1600 rpm in O_2–saturated 0.1 M KOH at 10 mV s^{-1}. The potential of the Pt ring was fixed at 0.5 V. The HO_2^- (%) and the electron transfer number (n) were determined by the following equations:

$$HO_2^- \,(\%) = 200 \times \frac{\mathrm{I_r/N}}{\mathrm{I_d} + \mathrm{I_r/N}}$$

$$n = 4 \times \frac{\mathrm{I_d}}{\mathrm{I_d} + \mathrm{I_r/N}}$$

where I_d is the disk current, I_r is the ring current and N is the current collection efficiency of the Pt ring. N was determined to be 0.37 from the reduction of $K_3[Fe(CN)_6]$.

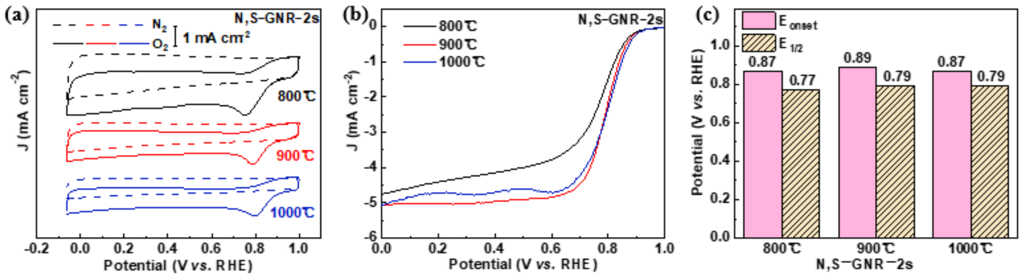

Figure S1. Electrochemical characterizations of N, S−GNR−2s synthesized at different temperatures in 0.1 M KOH. (a) CV curves in O_2−saturated and N_2−saturated solution. (b) RDE polarization curves at 1600 rpm. (c) Onset potentials and half−wave potentials.

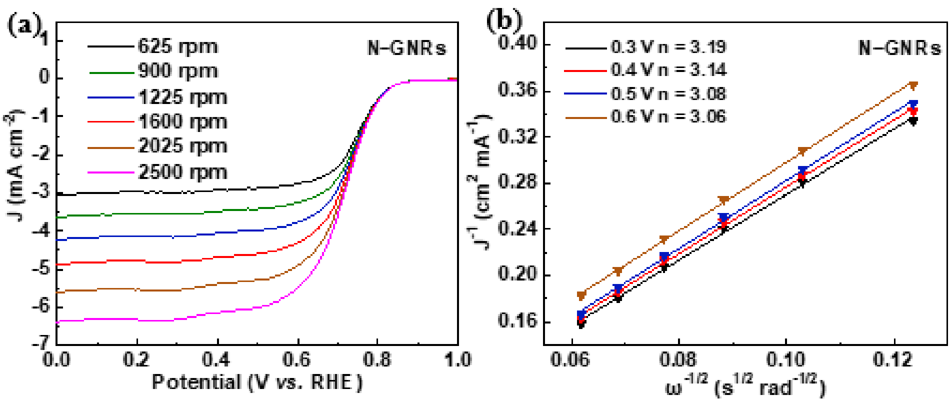

Figure S2. Electrochemical characterizations of N−GNRs. (a) RDE polarization curves at different rotating speeds in 0.1 M KOH and (b) the corresponding K–L plots at different potentials.

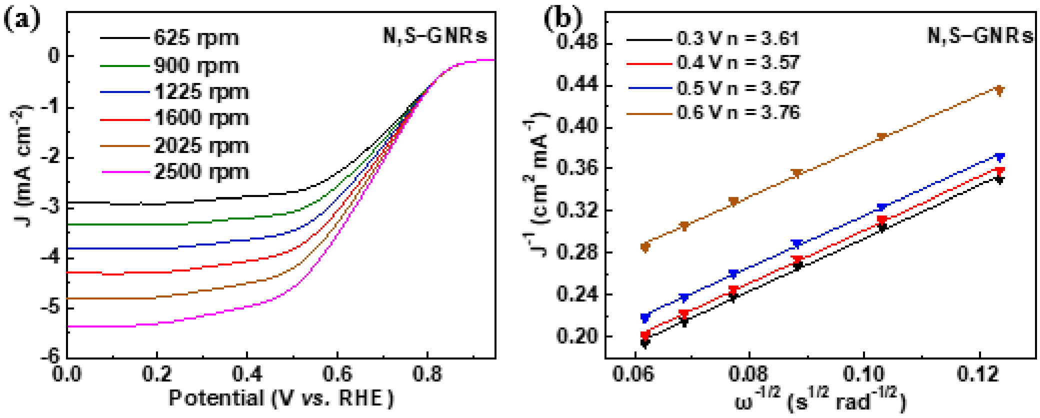

Figure S3. Electrochemical characterizations of N, S−GNRs. (a) RDE polarization curves at different rotating speeds in 0.1 M KOH and (b) the corresponding K–L plots at different potentials.

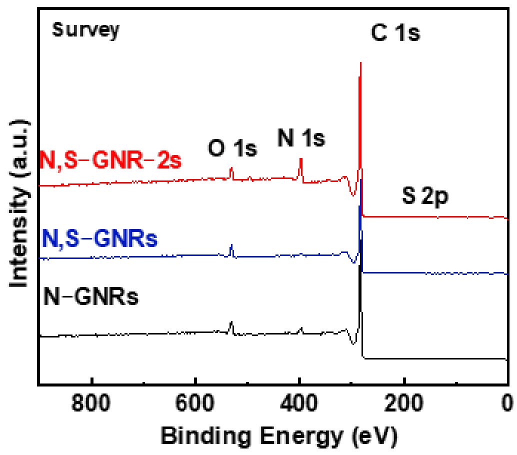

Figure S4. An XPS survey of N−GNRs, N, S−GNRs and N, S−GNR−2s.

Table S1. An electrocatalytic property comparison between our work and some relevant studies.

Materials	E_{onset}	$E_{1/2}$	n	References
N, S−GNR−2s	0.89 V vs. RHE	0.79 V vs. RHE	3.66 − 3.67	This work
N, S co−doped hierarchically porous carbon	0.84 V vs. RHE	0.74 V vs. RHE	–	[47]
N, S co−doped mesoporous dominated carbons	0.796 V vs. RHE	0.613 V vs. RHE	3.76 − 4.02	[48]
N/S co−doped multi−walled carbon nanotubes	0.87 V vs. RHE	0.71 V vs. RHE	3.8 − 3.9	[49]
N, S co−doped porous carbons	0.913 V vs. RHE	0.829 V vs. RHE	3.91 − 3.98	[50]
N, S co−doped graphene	0.002 V vs. Hg/HgO	–	2.98 − 3.36	[51]
N, S co−doped CNT−graphene hybrids	0.94 V vs. RHE	0.76 V vs. RHE	–	[52]
N, S co−doped oxygen functionalized carbons	0.92 V vs. RHE	0.81 V vs. RHE	3.9	[53]
N, S co−doped hierarchical porous carbon nanospheres	1.01 V vs. RHE	0.85 V vs. RHE	–	[54]
N, S−doped carbon framework	–	0.86 V vs. RHE	3.86 − 3.96	[55]

Table S2. The elemental contents of N−GNRs, N, S−GNRs and N, S−GNR−2s based on XPS analysis in Figure S4.

Materials	C %	N %	O %	S %
N−GNRs	88.81	5.43	5.76	–
N, S−GNRs	89.80	5.67	3.33	1.20
N, S−GNR−2s	84.66	10.93	3.40	1.01

Supplementary References

43. Li, W.; Min, C.G.; Tan, F.; Li, Z.P.; Zhang, B.S.; Si, R.; Xu, M.; Kiu, W.; Zhou, L.; Yang, X.K., Bottom−Up Construction of Active Sites in a Cu−N_4−C Catalyst for Highly Efficient Oxygen Reduction Reaction. *ACS Nano* **2019**, *13*, 3177–3187.
47. Li, R.R.; Liu, F.; Zhang, Y.H.; Guo, M.M.; Liu, D. Nitrogen, Sulfur Co−Doped Hierarchically Porous Carbon as a Metal−Free Electrocatalyst for Oxygen Reduction and Carbon Dioxide Reduction Reaction. *ACS Appl. Mater. Inter.* **2020**, *12*, 44578–44587.
48. Liu, J.T.; Wei, L.L.; Wang, H.Q.; Lan, G.J.; Yang, H.J.; Shen, J.Q. In−situ synthesis of heteroatom co−doped mesoporous dominated carbons as efficient electrocatalysts for oxygen reduction reaction. *Electrochim. Acta* **2020**, *364*, 11137335.
49. Patil, I.M.; Reddy, V.; Lokanathan, M.; Kakade, B. Nitrogen and Sulphur co−doped multiwalled carbon nanotubes as an efficient electrocatalyst for improved oxygen electroreduction. *Appl. Surf. Sci.* **2018**, *449*, 697–704.
50. Li, Y.Q.; Xu, H.B.; Huang, H.Y.; Gao, L.G.; Zhao, Y.Y.; Ma, T.L. Facile synthesis of N, S co−doped porous carbons from a dual−ligand metal organic framework for high performance oxygen reduction reaction catalysts. *Electrochim. Acta* **2017**, *254*, 148–154.
51. Zhang, H.H.; Liu, X.Q.; He, G.L.; Zhang, X.X.; Bao, S.J.; Hu, W.H. Bioinspired synthesis of nitrogen/sulfur co−doped graphene as an efficient electrocatalyst for oxygen reduction reaction. *J. Power Sources* **2015**, *279*, 252–258.
52. Huang, B.B.; Hu, X.; Liu, Y.C.; Qi, W.; Xie, Z.L. Biomolecule−derived N/S co−doped CNT−graphene hybrids exhibiting excellent electrochemical activities. *J. Power Sources* **2019**, *413*, 408–417.
53. Wang, S.T.; Liu, Y.; Liu, X.P.; Chen, Y.; Zhao, Y.L.; Gao, S.Y., Fabricating N, S Co−Doped Hierarchical Macro−Meso−Micro Carbon Materials as pH−Universal ORR Electrocatalysts. *ChemistrySelect* **2022**, *7*, e202200044.
54. Zhang, X.R.; Wang, Y.Q.; Du, Y.H.; Qing, M.; Yu, F.; Tian, Z.Q.; Shen, P.K. Highly active N,S co−doped hierarchical porous carbon nanospheres from green and template−free method for super capacitors and oxygen reduction reaction. *Electrochim. Acta* **2019**, *318*, 272–280.
55. Nong, J.; Zhu, M.; He, K.; Zhu, A.S.; Xie, P.; Rong, M.Z.; Zhang, M.Q. N/S co−doped 3D carbon framework prepared by a facile morphology−controlled solid−state pyrolysis method for oxygen reduction reaction in both acidic and alkaline media. *J. Energy Chem.* **2019**, *34*, 220–226.
